# Avidity of autoantibodies against type I interferons as a factor associated with neurological complications in severe COVID-19

**DOI:** 10.3389/fimmu.2026.1893615

**Published:** 2026-07-22

**Authors:** Mario Framil, Lydia García-Serrano, Adrián García-García, Inés Caballero-Segarra, Jose Luis Gomez-Vazquez, Arnau Antolí, Xavier Solanich

**Affiliations:** 1 Immunology Department, Centre Diagnòstic Biomèdic, Hospital Clínic de Barcelona, Barcelona, Spain; 2 Universitat de Barcelona, Barcelona, Spain; 3 Immunology Department, Hospital Universitari de Bellvitge, L’Hospitalet de Llobregat, Spain; 4 Bellvitge Biomedical Research Institute (IDIBELL), L’Hospitalet de Llobregat, Spain; 5 Internal Medicine Department, Hospital Universitari de Bellvitge, L’Hospitalet de Llobregat, Spain

**Keywords:** antibody avidity, anti-type I IFN autoantibodies, COVID-19, IFN-α, IFN-ω, neurological complications, neutralizing autoantibodies, Type I interferons

## Abstract

**Background:**

Autoantibodies against type I interferons (IFNs), particularly IFN-α2 and IFN-ω, are a recognized risk factor for severe COVID-19. Their functional characterization has largely focused on their neutralizing capacity. However, antibody binding avidity represents a distinct biophysical parameter that may be physiopathologically relevant. Whether avidity contributes to clinical outcomes in patient cohorts has not been evaluated.

**Methods:**

We characterized the avidity of IgG autoantibodies against IFN-α2 and IFN-ω by urea-dissociation ELISA in 95 patients with severe COVID-19 and confirmed anti-type I IFN autoantibodies. Avidity was classified as high (avidity index > 0.6) or low (avidity index ≤ 0.6). Clinical outcomes were compared between avidity groups, and patients were additionally stratified by combined avidity and neutralization profiles. Concordance between antibody subtypes was assessed by Cohen’s kappa.

**Results:**

Among 95 patients, 75 were positive for anti-IFN-α2 autoantibodies (51 high, 24 low avidity) and 49 for anti-IFN-ω autoantibodies (31 high, 18 low avidity). Low anti-IFN-α2 avidity was significantly associated with neurological complications (6/24 [25%] vs. 3/51 [6%]; OR 0.21 [0.05–0.84]; p = 0.026). For anti-IFN-ω, a non-significant trend in the same direction was observed (3/18 [17%] vs. 2/31 [6%]; p = 0.342). Avidity and neutralizing capacity were related but not interchangeable, with a low positive predictive value of avidity for neutralization (47% for anti-IFN-α2, 16% for anti-IFN-ω). Stratification by the combined avidity/neutralization phenotype identified the low-avidity + non-neutralizing combination of anti-IFN-α2 autoantibodies as the subgroup with the highest neurological complication rate (6/18 [33%]). Concordance between anti-IFN-α2 and anti-IFN-ω avidity was modest (Cohen’s κ = 0.24; 62% concordant).

**Conclusion:**

Antibody avidity is a functional characteristic of anti-type I IFN autoantibodies associated with neurological complications in severe COVID-19. In our cohort, avidity and neutralizing capacity are related but not equivalent, as a substantial proportion of high-avidity antibodies lack neutralizing activity, and the modest concordance between subtypes further suggests that these two autoantibody responses are partially independent. The combined assessment of avidity and neutralization identifies patients at higher neurological risk than either parameter alone, supporting avidity as an additional informative parameter in the functional characterization of anti-type I IFN autoantibodies.

## 1 Introduction

Type I interferons (IFNs) are central mediators of innate antiviral immunity. Upon viral detection, they signal through a shared heterodimeric receptor (IFNAR1/IFNAR2) to induce the expression of hundreds of interferon-stimulated genes that collectively restrict viral replication and coordinate adaptive immune responses (1–5). The clinical relevance of this pathway is underscored by data from the COVID-19 pandemic, which revealed that neutralizing autoantibodies against type I IFNs, particularly IFN-α2 and IFN-ω, were present in approximately 10–15% of patients with life-threatening COVID-19 pneumonia, far exceeding their prevalence in the general population (1, 6–8). These autoantibodies independently predicted intensive care admission, mechanical ventilation, and increased mortality (1, 6, 9–14).

However, not all anti-type I IFN autoantibodies are functionally or physiologically equivalent. Neutralizing antibodies, defined by their capacity to bind the cytokine, block its function, and suppress type I IFN–induced gene expression, account for only a fraction of all detectable autoantibodies. Non-neutralizing autoantibodies, which represent over 50% of detectable antibodies in several cohorts (2, 10, 15), have also been shown to exert pathological effects through distinct mechanisms. In systemic lupus erythematosus, non-neutralizing anti-IFN-α2 antibodies have been associated with elevated circulating cytokine levels and increased cardiovascular risk, likely by stabilizing IFN-α in immune complexes and extending its circulating half-life (16, 17). In severe COVID-19 patients, we have previously reported that non-neutralizing anti-type I IFN autoantibodies were significantly associated with thrombotic complications, potentially through a related mechanism whereby immune complex formation may prolong IFN bioavailability and sustain endothelial activation (18). These observations collectively highlight the importance of characterizing anti-IFN autoantibodies beyond their neutralizing capacity.

Antibody avidity, the overall binding strength of a multivalent antibody–antigen interaction, is a well-established parameter in clinical serology, where urea-dissociation assays are routinely used to distinguish recent from past infections such as cytomegalovirus and toxoplasmosis (19–21). A recent study by Groen et al. provided the first systematic characterization of the relationship between avidity and anti-IFN-I autoantibody function, showing that neutralizing anti-IFN-α2 and anti-IFN-ω autoantibodies consistently display high IgG avidities, whereas non-neutralizing autoantibodies tend to have lower avidities and can only partially block interaction with one of the two receptor subunits (22).

Neurological complications represent one of the most relevant manifestations of severe COVID-19, affecting 10–15% of hospitalized patients and encompassing a broad spectrum of disorders, from encephalopathy and stroke to peripheral neuropathy and new-onset seizures (23–26). Their pathogenesis is multifactorial, but emerging evidence implicates dysregulation of type I IFN signaling in neuroinflammatory processes (27–29). Whether anti-type I IFN autoantibodies contribute specifically to neurological risk, and through what functional mechanism, remains poorly defined.

In the present study, we characterized the avidity of IgG autoantibodies against IFN-α2 and IFN-ω in a well-characterized cohort of patients with severe COVID-19 previously screened for anti-type I IFN autoantibodies (10, 30). We aimed to determine whether avidity contributes to the clinical phenotype of these patients beyond what is captured by neutralizing activity, and to characterize the combined impact of avidity and neutralization on the risk of specific complications.

## 2 Materials and methods

### 2.1 Study design and patient population

This retrospective observational study was conducted in a previously described cohort of patients admitted to Hospital Universitari de Bellvitge (Barcelona, Spain) with severe COVID-19 pneumonia between April 2021 and December 2022. In our previous study, all patients were systematically screened for IgG autoantibodies against IFN-α2 and IFN-ω by ELISA, and their neutralizing activity was evaluated by a dual-luciferase reporter assay (30). For the present study, inclusion criteria were: (i) confirmed positivity for anti-IFN-α2 and/or anti-IFN-ω autoantibodies as defined in the parent cohort; and (ii) availability of sufficient residual serum for avidity measurement. Patients without sufficient residual serum were not included.

Clinical data were collected from prospectively maintained electronic medical records and anonymized before analysis, in accordance with Spanish data protection legislation (Ley Orgánica 3/2018 of 5 December). The study was approved by the Ethics Committee of Hospital Universitari de Bellvitge (reference PR40/21). All participants provided written informed consent. Serum samples were obtained from the HUB-ICO-IDIBELL Biobank.

### 2.2 Clinical variables and outcomes

Demographic data, comorbidities, vaccination status against SARS-CoV-2, and baseline pharmacological treatments were recorded at admission. Disease severity was assessed by the WHO ordinal scale at admission. Time from symptom onset to hospital admission was recorded in days. In-hospital treatments (remdesivir, tocilizumab, corticosteroids) and clinical complications were prospectively defined, including neurological complications (encephalopathy, stroke, peripheral neuropathy, new-onset seizures, or other documented neurological events), thrombotic complications, nosocomial infection, sepsis, septic shock, multiorgan failure, and in-hospital mortality. Duration of hospital stay was recorded in days.

### 2.3 Detection of anti-type I IFN autoantibodies

Autoantibodies against IFN-α2 and IFN-ω were detected by ELISA as previously described (10). Briefly, NUNC MaxiSorp™ plates were coated with recombinant human IFN-α2 or IFN-ω (1 mg/L) overnight at 4 °C, blocked with 5% non-fat milk in PBS, and incubated with 1:50 diluted serum samples in HPE buffer (Sanquin). Fc-specific HRP-conjugated anti-human IgG (Nordic-MUbio) was used as the secondary antibody. Samples were considered positive when optical density at 450 nm exceeded the mean plus two standard deviations of a healthy control reference group.

### 2.4 Neutralization assay

Neutralizing activity was evaluated using a dual-luciferase reporter assay as previously described (10). Briefly, HEK293T cells were transfected with a firefly luciferase construct under ISRE promoter control, together with constitutive Renilla luciferase for normalization, and incubated with patient serum at 1:10 dilution in the presence of recombinant IFN-α2 or IFN-ω at 10 ng/mL for 24 h at 37 °C in 5% CO_2_. Antibodies reducing the normalized signal below 15% of the negative-control mean were classified as neutralizing.

### 2.5 Avidity assay

Antibody avidity was determined using a modified urea-dissociation ELISA based on the standard detection protocol. Following 2 h of serum incubation at 1:100 dilution, wells were washed either with PBS-Tween (standard condition) or with urea in PBS at three concentrations (4 M, 6 M, and 8 M) for 12 min at 22 ± 2 °C under continuous agitation. The avidity index (AI) at each urea concentration was calculated as the ratio of optical density (OD) after urea treatment to OD under the standard (PBS-Tween only) condition: AI = OD_urea/OD_standard, yielding a value of 1.0 when no dissociation occurs. The three concentrations were tested in all samples to characterize the concentration-dependent dissociation profile (**Supplementary Figure 1**). The AI at 6 M urea was used for group classification, as this concentration provided the clearest separation between high- and low-avidity strata across both antibody subtypes. Antibodies were classified as low avidity when AI ≤ 0.6 and as high avidity when AI > 0.6 at 6 M urea, following thresholds used in established avidity assays for cytokine and viral serology (31, 32).

### 2.6 Statistical analysis

Continuous variables are reported as median [interquartile range, IQR] and compared by two-tailed Mann-Whitney U test. Effect sizes for continuous comparisons were quantified by Cliff’s delta (δ). Categorical variables are expressed as n (%) and compared by Fisher’s exact test. Odds ratios (OR) with 95% confidence intervals (CI) were estimated from 2×2 contingency tables. Differences in event proportions across the four phenotypic subgroups (avidity × neutralization) were compared using the Fisher’s exact test for r×c contingency tables. Odds ratios were computed with the Haldane–Anscombe continuity correction, and 95% confidence intervals derived from the Wald approximation on the log-odds scale. Two-sided p-values were obtained from Fisher’s exact test. Concordance between anti-IFN-α2 and anti-IFN-ω avidity was assessed by Cohen’s kappa (κ). The predictive performance of high avidity for neutralizing activity was characterized by positive predictive value [PPV = true positives/(true positives + false positives)] and negative predictive value [NPV = true negatives/(true negatives + false negatives)], considering high avidity as the predictor and the presence of neutralizing activity as the reference. A p-value < 0.05 was considered significant. Analyses were performed using Python 3.12 (NumPy 1.26, SciPy 1.12, Pandas 2.1). The continuous correlation between avidity index and neutralization capacity was assessed by Spearman’s rank correlation.

## 3 Results

### 3.1 Study population and avidity classification

Out of 95 patients with severe COVID-19 and confirmed anti-type I IFN autoantibodies, 75 (79%) were positive for anti-IFN-α2 and 49 (52%) for anti-IFN-ω. Among anti-IFN-α2-positive patients, 51 (68%) were classified as high avidity and 24 (32%) as low avidity; among anti-IFN-ω-positive patients, 31 (63%) were high avidity and 18 (37%) low avidity. In the 29 patients positive for both autoantibodies, 10 (34%) had concordant high avidity for both subtypes, 8 (28%) had concordant low avidity, and 11 (38%) showed discordant profiles. Concordance between anti-IFN-α2 and anti-IFN-ω avidity was modest (Cohen’s κ = 0.24; 18/29, 62% concordant). The overlap between anti-IFN-α2 and anti-IFN-ω positivity, together with the corresponding avidity and neutralization profiles, is shown in **Supplementary Figure 2**.

### 3.2 Baseline characteristics according to anti-IFN-α2 and anti-IFN-ω avidity

Baseline characteristics are shown in **Table 1** for the anti-IFN-α2 cohort and the anti-IFN-ω cohort. Within the anti-IFN-α2 cohort, the high- and low-avidity groups were well matched for age (74 [60–86] vs. 77 [60–86] years; p = 0.578), sex (61% vs. 50% male; p = 0.456), and all major comorbidities. Disease severity at admission was identical [WHO ordinal scale 4 (4–5) vs. 4 (4–5); p = 0.936]. The only significant difference in admission characteristics was the interval from symptom onset to hospitalization, which was longer in the high-avidity group [median 7 (4–10) vs. 5 (3–7) days; Cliff’s δ = 0.284 (95% CI 0.011–0.534); p = 0.048]. Vaccination rates did not differ (82% vs. 88%; p = 0.741), and in-hospital treatments were comparable between groups.

**Table 1:** Baseline demographic, clinical, and treatment characteristics of patients with severe COVID-19 stratified by anti-IFN-α2 and anti-IFN-ω autoantibody avidity.

Variable	anti-IFN-α2 avidity				anti-IFN-ω avidity			
	Overall (n=75)	High (n=51)	Low (n=24)	p	Overall (n=49)	High (n=31)	Low (n=18)	p
*Demographics*								
Age (years), median [IQR]	75 [60–86]	74 [60–86]	77 [60–86]	0.578	71 [56–86]	67 [52–76]	76 [65–87]	0.044*
Male sex, n (%)	43 (57%)	31 (61%)	12 (50%)	0.456	28 (57%)	19 (61%)	9 (50%)	0.553
*Comorbidities*								
Obesity, n (%)	19 (25%)	13 (25%)	6 (25%)	1.000	11 (22%)	9 (29%)	2 (11%)	0.178
Current smoker, n (%)	7 (9%)	3 (6%)	4 (17%)	0.201	4 (8%)	1 (3%)	3 (17%)	0.134
Pulmonary disease, n (%)	23 (31%)	15 (29%)	8 (33%)	0.791	19 (39%)	11 (35%)	8 (44%)	0.559
Diabetes mellitus, n (%)	18 (24%)	14 (27%)	4 (17%)	0.392	13 (27%)	7 (23%)	6 (33%)	0.508
Arterial hypertension, n (%)	50 (67%)	34 (67%)	16 (67%)	1.000	23 (47%)	16 (52%)	7 (39%)	0.554
Dyslipidemia, n (%)	28 (37%)	19 (37%)	9 (38%)	1.000	20 (41%)	13 (42%)	7 (39%)	1.000
Cardiac disease, n (%)	17 (23%)	9 (18%)	8 (33%)	0.149	11 (22%)	7 (23%)	4 (22%)	1.000
Renal disease, n (%)	16 (21%)	11 (22%)	5 (21%)	1.000	7 (14%)	4 (13%)	3 (17%)	0.697
Hepatic disease, n (%)	3 (4%)	3 (6%)	0 (0%)	0.547	2 (4%)	2 (6%)	0 (0%)	0.526
Active cancer, n (%)	12 (16%)	7 (14%)	5 (21%)	0.505	12 (24%)	9 (29%)	3 (17%)	0.494
Immunodeficiency, n (%)	7 (9%)	5 (10%)	2 (8%)	1.000	5 (10%)	4 (13%)	1 (6%)	0.639
Baseline steroids/IS, n (%)	5 (7%)	3 (6%)	2 (8%)	0.653	4 (8%)	3 (10%)	1 (6%)	1.000
ACEi or ARB use, n (%)	27 (36%)	20 (39%)	7 (29%)	0.449	13 (27%)	10 (32%)	3 (17%)	0.322
Anticoagulation (baseline), n (%)	9 (12%)	5 (10%)	4 (17%)	0.455	8 (16%)	5 (16%)	3 (17%)	1.000
*Admission parameters*								
COVID-19 vaccination, n (%)	63 (84%)	42 (82%)	21 (88%)	0.741	37 (76%)	20 (65%)	17 (94%)	0.036*
Days symptoms to admission, median [IQR]	6 [4–10]	7 [4–10]	5 [3–7]	0.048*	6 [3–8]	6 [4–8]	5 [3–10]	0.950
WHO ordinal scale, median [IQR]	4 [4–5]	4 [4–5]	4 [4–5]	0.936	4 [4–5]	4 [4–5]	4 [4–4]	0.752
*In-hospital treatment*								
Remdesivir, n (%)	23 (31%)	15 (29%)	8 (33%)	0.791	18 (37%)	10 (32%)	8 (44%)	0.540
Tocilizumab, n (%)	17 (23%)	11 (22%)	6 (25%)	0.773	5 (10%)	5 (16%)	0 (0%)	0.143
Corticosteroids (treatment), n (%)	66 (88%)	44 (86%)	22 (92%)	0.710	43 (88%)	27 (87%)	16 (89%)	1.000

Within the anti-IFN-ω cohort (**Table 1**), high-avidity patients were significantly younger [67 (52–76) vs. 76 (65–87) years; p = 0.044] and had a significantly lower COVID-19 vaccination rate (65% vs. 94%; p = 0.036). No other baseline variables differed.

### 3.3 Clinical outcomes according to anti-IFN-α2 and anti-IFN-ω avidity

**Figure 1:**
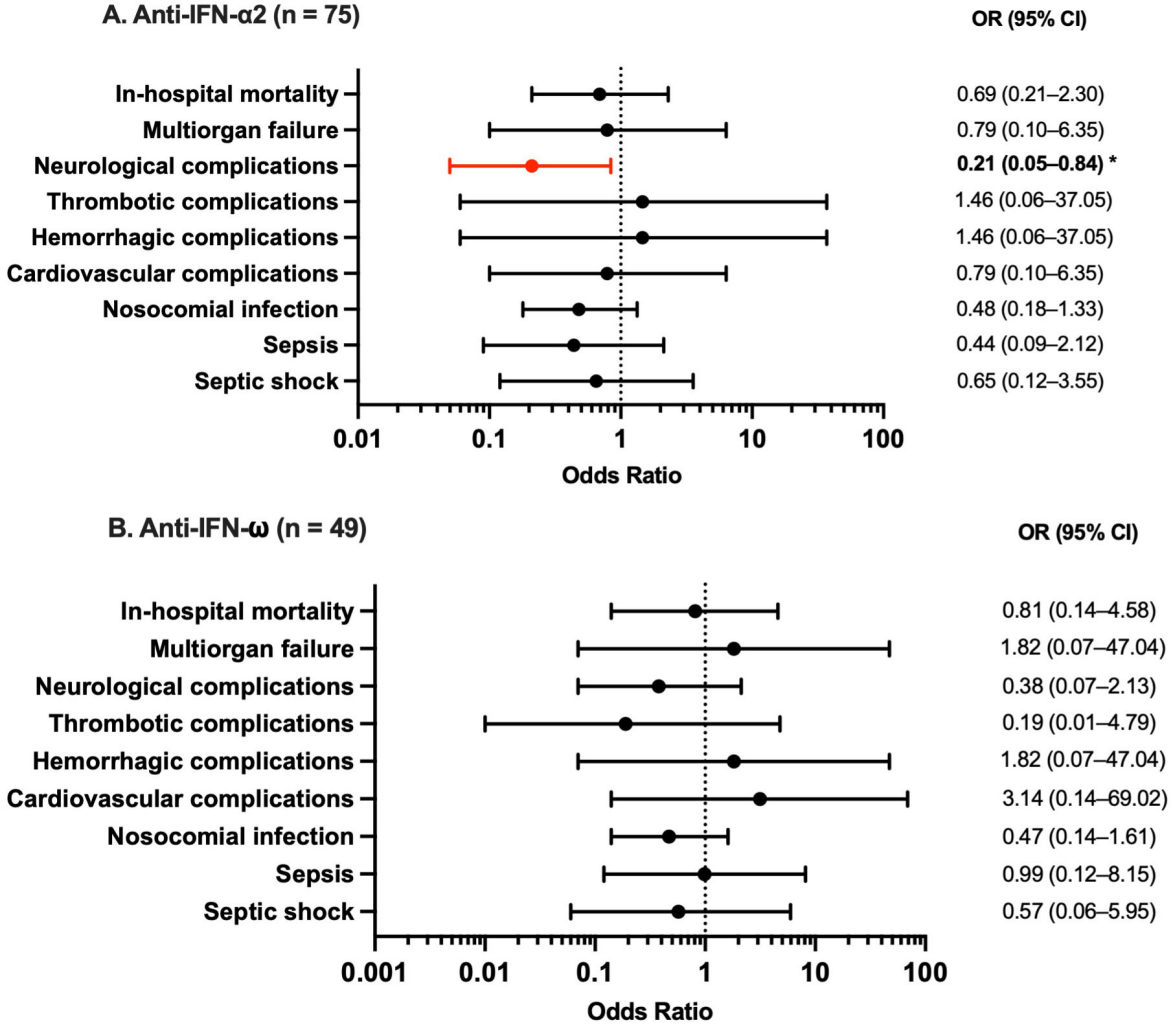
Forest plot of unadjusted odds ratios (ORs) for binary clinical outcomes according to anti-IFN-α2 avidity (high vs. low; panel **(A)** n = 75) and anti-IFN-ω avidity (high vs. low; panel **(B)** n = 49). ORs < 1 indicate lower event rates in high-avidity patients (reference: low avidity). Error bars represent 95% confidence intervals. The dashed vertical line indicates OR = 1 (no effect). The key finding, neurological complications in anti-IFN-α2 (OR 0.21 [0.05–0.84]; p = 0.026), is shown in red. All other comparisons were non-significant. Note the logarithmic x-axis scale.

Clinical outcomes are detailed in **Table 2**. In the anti-IFN-α2 cohort, in-hospital mortality was comparable between avidity groups [8/51 (16%) vs. 5/24 (21%); OR 0.69 (0.21–2.30); p = 0.745], as were the WHO ordinal scale, hospital stay, and all non-neurological complications. Forest plots of all outcome comparisons are presented in **Figure 1**. Neurological complications occurred significantly more frequently in patients with low anti-IFN-α2 avidity [6/24 (25%) vs. 3/51 (6%); OR 0.21 (0.05–0.84); p = 0.026]. Notably, 8 of the 9 documented neurological events corresponded to acute confusional syndromes, consistent with encephalopathy. For anti-IFN-ω (**Table 2**), no statistically significant differences were observed for any clinical endpoint, including neurological complications [2/31 (6%) vs. 3/18 (17%); OR 0.38 (0.07–2.13); p = 0.342], in-hospital mortality [3/31 (10%) vs. 2/18 (11%); p = 1.000], and hospital stay [11 (6–15) vs. 8 (5–12) days; p = 0.224]. A non-significant trend toward lower neurological complication rates was observed in the high-avidity group, in the same direction as observed for anti-IFN-α2.

**Table 2:** Clinical outcomes of patients with severe COVID-19 stratified by anti-IFN-α2 and anti-IFN-ω autoantibody avidity.

Variable	anti-IFN-α2 avidity				anti-IFN-ω avidity			
	Overall (n=75)	High (n=51)	Low (n=24)	p	Overall (n=49)	High (n=31)	Low (n=18)	p
Hospital stay (days), median [IQR]	9 [6–14]	10 [6–18]	8 [6–12]	0.285	9 [6–13]	11 [6–15]	8 [5–12]	0.224
In-hospital mortality, n (%)	13 (17%)	8 (16%)	5 (21%)	0.745	5 (10%)	3 (10%)	2 (11%)	1.000
Multiorgan failure, n (%)	3 (4%)	2 (4%)	1 (4%)	1.000	1 (2%)	1 (3%)	0 (0%)	1.000
Neurological complications, n (%)	9 (12%)	3 (6%)	6 (25%)	0.026*	5 (10%)	2 (6%)	3 (17%)	0.342
Thrombotic complications, n (%)	1 (1%)	1 (2%)	0 (0%)	1.000	1 (2%)	0 (0%)	1 (6%)	0.367
Hemorrhagic complications, n (%)	1 (1%)	1 (2%)	0 (0%)	1.000	1 (2%)	1 (3%)	0 (0%)	1.000
Cardiovascular complications, n (%)	3 (4%)	2 (4%)	1 (4%)	1.000	2 (4%)	2 (6%)	0 (0%)	0.526
Cardiorespiratory arrest, n (%)	1 (1%)	1 (2%)	0 (0%)	1.000	0 (0%)	0 (0%)	0 (0%)	—
Nosocomial infection, n (%)	23 (31%)	13 (25%)	10 (42%)	0.185	14 (29%)	7 (23%)	7 (39%)	0.326
Sepsis, n (%)	6 (8%)	3 (6%)	3 (12%)	0.377	3 (6%)	2 (6%)	1 (6%)	1.000
Septic shock, n (%)	5 (7%)	3 (6%)	2 (8%)	0.653	2 (4%)	1 (3%)	1 (6%)	1.000

### 3.4 Relationship between avidity and neutralizing capacity

We examined the relationship between avidity and neutralizing capacity for anti-IFN-α2 and anti-IFN-ω autoantibodies. In the anti-IFN-α2 cohort, 30/75 patients (40%) had neutralizing antibodies. Neutralizing activity was more frequent in the high-avidity group than in the low-avidity group [24/51 (47%) vs. 6/24 (25%); p = 0.082]. However, high avidity did not necessarily imply neutralization, as 27/51 patients (53%) with high-avidity antibodies lacked neutralizing activity.

In contrast, for anti-IFN-ω, neutralizing antibodies were detected in 8/49 patients (16%) and were similarly distributed between avidity groups [5/31 (16%) in the high-avidity group vs. 3/18 (17%) in the low-avidity group; p = 1.000]. The positive predictive value of high avidity for neutralizing activity was 47% for anti-IFN-α2 and 16% for anti-IFN-ω; negative predictive values were 75% and 83%, respectively.

When patients were stratified solely by neutralizing activity, neurological complications occurred in 2/30 (6.7%) neutralizing vs. 7/45 (15.6%) non-neutralizing anti-IFN-α2 patients (p = 0.301), and in 0/8 (0%) vs. 5/41 (12.2%) for anti-IFN-ω (p = 0.575). Neither comparison reached statistical significance.

The avidity index showed a weak correlation with the neutralization assay for anti-IFN-α2 autoantibodies (Spearman’s ρ = −0.347, p = 0.002) and no significant correlation for anti-IFN-ω autoantibodies (Spearman’s ρ = −0.099, p = 0.497).

### 3.5 Stratification by avidity and neutralizing capacity

**Figure 2:**
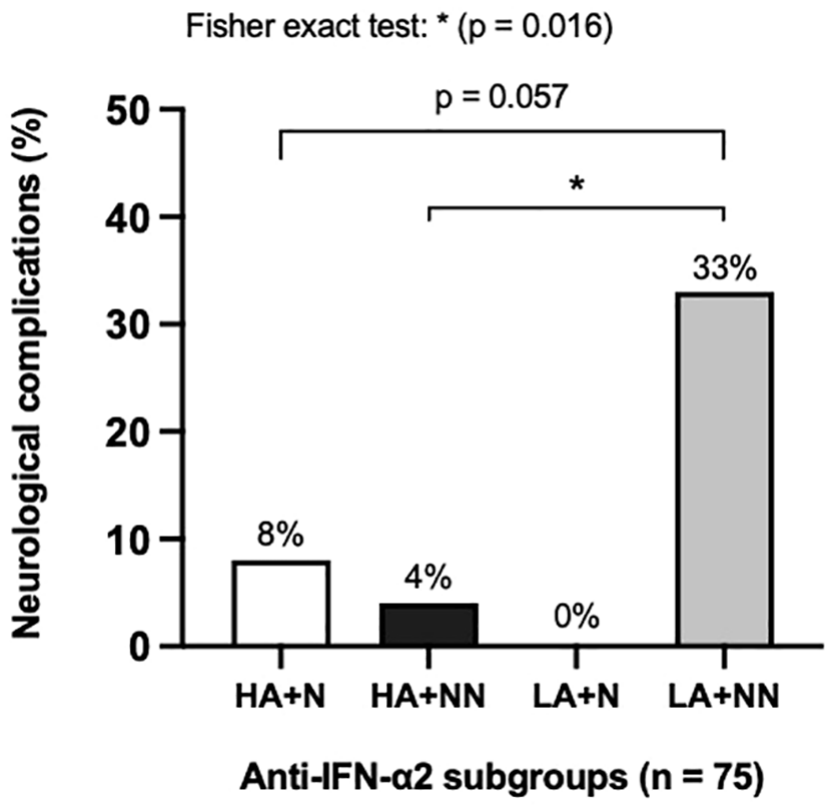
Neurological complication rates across the four phenotypic subgroups defined by the combination of anti-IFN-α2 avidity (high vs. low) and neutralizing capacity (neutralizing vs. non-neutralizing). The percentage in each bar represents the proportion of patients with at least one neurological complication. Numbers in parentheses denote subgroup sizes. The low-avidity + non-neutralizing subgroup (LA+NN) exhibited the highest neurological risk (6/18; 33%). Global Fisher’s exact test across the four subgroups: p = 0.016.

To examine the combined contributions of anti-IFN-α2 avidity and neutralizing capacity to clinical outcomes, patients were classified into four subgroups: high avidity + neutralizing (n = 24), high avidity + non-neutralizing (n = 27), low avidity + neutralizing (n = 6), and low avidity + non-neutralizing (n = 18). Neurological complication rates were 2/24 (8%), 1/27 (4%), 0/6 (0%), and 6/18 (33%), respectively (**Figure 2**), with the low-avidity + non-neutralizing combination showing the highest rate of events (Fisher’s exact test, p = 0.016). The pairwise comparison vs. the high-avidity + non-neutralizing subgroup was statistically significant (p = 0.012). A parallel stratification of the anti-IFN-ω cohort showed neurological complication rates of 0/5 (0%), 2/26 (8%), 0/3 (0%), and 3/15 (20%) across the four subgroups; the low-avidity + non-neutralizing subgroup again exhibited the highest rate, although none of the differences reached statistical significance. Among the 29 patients positive for both autoantibody types, concordant high avidity (n = 10) was associated with 0/10 (0%) neurological complications, compared with 3/8 (38%) in concordant low avidity (n = 8) (OR 0.07 [0.00–1.35]; p = 0.069); patients with discordant avidity profiles (n = 11) showed an intermediate rate (1/11; 9%).

Neurological complication rates were also compared with those of patients without anti-type I IFN autoantibodies from the source cohort [n = 566; 48 events (8.5%); ref. 30]. Anti-IFN-α2-positive patients overall showed no significant difference compared with this reference population [9/75 (12.0%); OR 1.47, p = 0.287]. By contrast, neurological complications were more frequent in the low-avidity subgroup [6/24 (25.0%); OR 3.60, p = 0.016] and in the low-avidity + non-neutralizing subgroup [6/18 (33.3%); OR 5.40, p = 0.004], whereas the high-avidity + neutralizing subgroup showed a similar rate to the reference population [2/24 (8.3%); OR 0.98, p = 1.000].

## 4 Discussion

In this study, we characterized the avidity of anti-IFN-α2 and anti-IFN-ω autoantibodies in patients with severe COVID-19 and examined its association with clinical outcomes. We found that low anti-IFN-α2 avidity is significantly associated with neurological complications, regardless of neutralizing capacity. Furthermore, combined stratification by avidity and neutralizing status identifies the low-avidity + non-neutralizing phenotype as the subgroup at highest neurological risk. These results suggest that avidity is a functionally relevant parameter for the clinical characterization of anti-type I IFN autoantibodies.

Our data complement the study by Groen et al. (22), which showed that neutralizing anti-IFN-I autoantibodies display high avidities and block the interaction of IFN-α and IFN-ω with both IFNAR1 and IFNAR2, whereas non-neutralizing autoantibodies tend to exhibit lower avidities and only partially interfere with receptor binding. In our cohort, neutralizing activity among anti-IFN-α2 autoantibodies was more frequent in the high-avidity group than in the low-avidity group [24/51 (47%) vs. 6/24 (25%); p = 0.082], suggesting a possible association between avidity and neutralizing capacity. However, this association was incomplete, as more than half of high-avidity patients [27/51 (53%)] lacked neutralizing activity. In contrast, for anti-IFN-ω autoantibodies, neutralizing activity was similarly distributed between avidity groups [5/31 (16%) vs. 3/18 (17%); p = 1.000], indicating no evident relationship between avidity and neutralization. Overall, these findings suggest that avidity and neutralizing capacity are related but not interchangeable in this cohort, with high avidity showing limited positive predictive value for neutralizing capacity. A plausible explanation, also supported by Groen et al. (22), is that some autoantibodies bind IFN-α2 with high avidity at epitopes that do not interfere with IFNAR engagement, forming stable immune complexes without preventing receptor signaling and thus dissociating binding strength from functional neutralization.

Several factors may account for the incomplete concordance between avidity and neutralization observed in our cohort. Compared with the patients studied by Groen et al., our cohort has two key distinguishing features: all patients had critical COVID-19 with a pronounced hyperinflammatory background, and a substantial proportion had received mRNA vaccination against SARS-CoV-2. These clinical characteristics may favor the emergence of low-avidity clones with distinct temporal dynamics. Consistent with this, Shaw et al. reported differential temporal dynamics for anti-IFN-α2 and anti-IFN-ω autoantibodies in COVID-19, with binding levels and neutralizing activity peaking during the acute phase and declining into convalescence, and partially independent kinetics between subtypes (33). These observations are relevant to our findings, as single time-point serum measurements, particularly during or shortly after the acute hyperinflammatory phase, may not fully capture the avidity landscape across the disease course. During the acute phase there is likely to be a broader mixture of polyclonal antibodies with diverse avidities, whereas at later time points the repertoire may be enriched for clones with higher avidity. The avidity index measured by urea-dissociation ELISA reflects the aggregate binding stability of the total IgG response, encompassing a mixture of high- and low-avidity clones with potentially distinct functional consequences.

In our cohort, avidity, and particularly the combination of avidity with neutralizing capacity, identifies a subset of patients with severe COVID-19 and anti-IFN autoantibodies who appeared to be at increased risk of neurological complications. Of note, 8 of the 9 neurological events in our cohort presented as acute confusional syndrome, a phenotype consistent with cytokine-mediated neuroinflammation. This observation is consistent with the differential capacity of antibodies with distinct avidities to modulate IFN–receptor interactions. High-avidity antibodies may form more stable immune complexes that effectively limit IFN bioavailability, whereas low-avidity antibodies may generate less stable complexes, resulting in a dynamic equilibrium that favors the presence of free IFN and allows intermittent receptor engagement and sustained signaling; mechanistic models of cytokine–antibody interactions support the notion that non-neutralizing or partially binding antibodies can paradoxically enhance cytokine bioavailability and signaling under certain conditions (34). In a similar way, non-neutralizing anti-IFN-α2 antibodies in systemic lupus erythematosus have been associated with higher circulating levels of IFN-α and with worse clinical outcomes, including cardiovascular events (16). In this context, low-avidity antibodies could promote dysregulated neuroinflammatory responses or facilitate the engagement of activating Fc receptors on brain-resident innate cells, although the specific mechanisms in the CNS remain to be elucidated.

Consistent with this model, our previous work on non-neutralizing autoantibodies (18) supports the concept that such antibodies can prolong the half-life of circulating IFN within immune complexes, thereby sustaining IFN signaling in tissue-specific contexts, including the vascular endothelium, where type I IFN can directly drive microvascular dysfunction (35).

Stratification by both avidity and neutralizing capacity supports the physiological model outlined above. The highest neurological complication rate was observed in the low-avidity + non-neutralizing subgroup [6/18 (33%)], while the two high-avidity subgroups (neutralizing and non-neutralizing) showed comparably low rates [2/24 (8%) and 1/27 (4%), respectively], and the low-avidity + neutralizing subgroup showed no neurological events [0/6 (0%)]. This pattern was further supported by comparison with patients without anti-type I IFN autoantibodies from the source cohort (30): the mere presence of anti-IFN-α2 autoantibodies was not by itself associated with increased neurological risk, whereas low avidity alone discriminated a subgroup at increased risk, and HA+N patients showed rates virtually identical to those of patients without autoantibodies (8.3% vs. 8.5%). Overall, these data support the complementary assessment of both parameters in the functional evaluation of anti-IFN autoantibodies.

The absence of statistically significant outcome differences for anti-IFN-ω likely reflects the limited sample size for this subtype (n = 49, 5 neurological events), rather than a true lack of biological effect. A consistent trend toward lower neurological complication rates in the high-avidity ω group [2/31 (6%) vs. 3/18 (17%)], together with the pattern observed in the combined both-high vs. both-low avidity analysis [0/10 (0%) vs. 3/8 (38%); p = 0.069], supports biological plausibility. The modest concordance between anti-IFN-α2 and anti-IFN-ω avidity (κ = 0.24) further suggests that these two autoantibody responses may be partially independent. Of note, the associations of anti-IFN-ω avidity with younger age and lower vaccination rates observed in our cohort warrant further investigation in larger and adequately powered cohorts.

Several limitations of this study should be acknowledged. First, the retrospective, single-center design limits causal inference and external generalizability, and external validation in independent cohorts will be essential. Second, sample sizes were modest, particularly for the anti-IFN-ω cohort and for outcomes with low event counts. Third, avidity was measured at a single time-point during the acute phase of disease, which may not fully capture the temporal evolution of the autoantibody response (33). Fourth, the avidity threshold applied in this study (AI > 0.6) was a predefined cut-off based on established avidity assay protocols and on a prior methodological optimization step in which this value provided the clearest discrimination between high- and low-avidity strata (**Supplementary Figure 1**). Fifth, additional analyses were limited by the residual serum available after the avidity, neutralization, and ELISA assays.

In conclusion, we provide evidence that antibody avidity represents a distinct functional characteristic of anti-type I IFN autoantibodies associated with neurological complications in severe COVID-19. In our cohort, avidity and neutralizing capacity are related but not equivalent: a substantial proportion of high-avidity antibodies lacked neutralizing activity, indicating that avidity captures biological information not inferred from neutralization assays alone. The combination of low avidity and absent neutralizing activity identifies a subset of patients at increased neurological risk. Together with our previous report on non-neutralizing autoantibodies and thrombotic complications (18), these findings underscore the relevance of comprehensive functional characterization of anti-type I IFN autoantibodies, with potential implications for clinical risk stratification and for improving our understanding of conditions in which non-neutralizing anti-cytokine autoantibodies are pathophysiologically relevant.

## Data Availability

The original contributions presented in the study are included in the article/**Supplementary Material**. Further inquiries can be directed to the corresponding author.

## Supplementary material

Supplementary Figure 1 Concentration-dependent avidity dissociation profiles for anti-IFN-α2 (panel A, n = 75) and anti-IFN-ω (panel B, n = 49) autoantibodies across three urea concentrations. The avidity index (AI) is shown as group mean ± standard deviation (symbols with vertical error bars), with values connected by lines for visual clarity, at PBS baseline (AI normalized to 1.0), 4 M, 6 M, and 8 M urea. Samples are grouped by their classification at 6 M urea: high avidity (AI > 0.6; anti-IFN-α2 n = 51, anti-IFN-ω n = 31) and low avidity (AI ≤ 0.6; anti-IFN-α2 n = 24, anti-IFN-ω n = 18). The dashed horizontal line indicates the AI = 0.6 classification threshold. In both panels, AI decreases progressively with increasing urea concentration, and the two groups remain clearly separated across all three dissociation conditions. For anti-IFN-α2, the proportion of samples exceeding the threshold was 83 % (62/75) at 4 M, 68 % (51/75) at 6 M, and 44 % (33/75) at 8 M, confirming that 6 M urea provides the best discrimination between avidity strata. Mean ± SD at 6 M: high avidity 0.759 ± 0.101 vs. low avidity 0.428 ± 0.146 (anti-IFN-α2); 0.757 ± 0.089 vs. 0.481 ± 0.085 (anti-IFN-ω). AI, avidity index; PBS, phosphate-buffered saline.

Supplementary Figure 2 Distribution of anti-IFN-α2 and anti-IFN-ω autoantibody positivity in the study cohort (n = 95), with the corresponding functional subgroups defined by avidity and neutralizing activity. Forty-six patients were positive only for anti-IFN-α2 autoantibodies, 20 only for anti-IFN-ω autoantibodies, and 29 patients were positive for both autoantibody subtypes (double-positive). The distribution of functional subgroups within each region is shown below the respective patient counts, with anti-IFN-α2 and anti-IFN-ω profiles indicated separately within the double-positive group. HA, high avidity; LA, low avidity; N, neutralizing; NN, non-neutralizing.
